# A Case-Control Study of Risk Factors for Salivary Gland Cancer in Canada

**DOI:** 10.1155/2017/4909214

**Published:** 2017-01-04

**Authors:** Sai Yi Pan, Margaret de Groh, Howard Morrison

**Affiliations:** Science Integration Division, Social Determinant and Science Integration Directorate, Public Health Agency of Canada, Ottawa, ON, Canada

## Abstract

*Aim*. To assess the effect of various lifestyle risk factors on the risk of salivary gland cancer in Canada using data from a population-based case-control study.* Methods*. Data from a population-based case-control study of 132 incident cases of salivary gland cancer and 3076 population controls were collected through self-administered questionnaire and analysed using unconditional logistic regression.* Results*. Four or more servings/week of processed meat product was associated with an adjusted odds ratio (OR) and corresponding 95% confidence interval (CI) of 1.62 (1.02–2.58). Nonsignificantly increased ORs were also related to obesity, >7 drinks/week of alcohol consumption, and occupational exposure to radiation. Furthermore, nonsignificantly decreased ORs were found to be associated with high education level (>12 years) (OR = 0.65), high consumption of spinach/squash (OR = 0.62) and all vegetables/vegetable juices (OR = 0.75), and >30 sessions/month of recreational physical activity (OR = 0.78).* Conclusions*. This study suggests positive associations with consumption of processed meat, smoking, obesity, alcohol drinking, and occupational exposure to radiation as well as negative associations with higher education, consumption of spinach/squash, and physical activity, which suggest a role of lifestyle factors in the etiology of salivary gland cancer. However, these findings were based on small number of cases and were nonsignificant. Further larger studies are warranted to confirm our findings.

## 1. Introduction

Salivary gland cancer arises in the three major salivary glands (parotid, sublingual, and submandibular) and 600–1000 minor salivary glands distributed throughout the aerodigestive tract, with most occurring in the parotid [1, 2]. Salivary gland cancer is rare; internationally, age-standardized incidence rates vary from 0.5 to 2.0 cases per 100,000 people per year [3]. In 2010, there were an estimated 450 new cases of salivary gland cancer in Canada: 260 males and 195 females. This corresponded to 11% of all oral cancers and 0.3% of all malignancies [4].

Because of its rareness, very little is known about the etiology of salivary gland cancers. A few studies have suggested that radiation exposure (e.g., radiation treatment to the head and neck area), certain workplace exposures (e.g., nickel alloy dust, silica dust, asbestos, mining, and rubber products manufacturing), tobacco use, alcohol consumption, diets low in vegetables and high in animal fat, and heavy cell phone use are possible risk factors [1, 5]. Also implicated has been a previous history of cancer [6] and exposure to certain viruses including HIV [7], Epstein Barr [8], and possibly HPV [9].

The current study assessed possible risk factors for primary salivary gland cancer in Canada using data from the Canadian National Enhanced Cancer Surveillance System (NECSS).

## 2. Materials and Methods

### 2.1. Study Population

The NECSS was a collaborative, multicenter, and multicomponent project between Health Canada and the provincial cancer registries, which aimed at evaluating the etiological role of environmental factors in various types of cancers. The case-control component included individual data from 21,020 Canadians with one of 19 types of cancers and 5,039 population controls aged 20 to 76 years collected between 1994 and 1997 in eight of ten Canadian provinces (Alberta, British Columbia, Manitoba, Newfoundland, Nova Scotia, Ontario, Prince Edward Island, and Saskatchewan). An ethics committee from each participating province reviewed and approved the study proposal. The current analysis was based on 132 incident cases of salivary gland cancer and 3076 population controls.

Incident primary salivary gland cancer cases were identified through pathology reports within a few months of diagnosis to reduce loss of subjects from death or severe illness. All cases included were confirmed histologically according to the* International Classification of Diseases for Oncology, Second Edition (ICD-O-2) *(142.0–142.9 and C08.0–C09.9). In total, the registries identified 307 cases of salivary gland cancer. Physicians did not provide consent to contact for 21 cases, and 37 cases died before questionnaires could be sent. Therefore, questionnaires were mailed to 250 cases. Of cases sent questionnaire, 132 completed and returned the questionnaire, representing 52.8% of cases sent questionnaires.

Control subjects were residents from the participating provinces with no prior diagnosis of cancer. The NECSS used frequency matching to the overall case group (all 19 types of cancer) to select population controls matched by five-year age group and sex within each province. Details of the recruitment methods and response rates for control subjects have been described elsewhere [10]. Questionnaires identical to the ones sent to cases were mailed to 8117 controls. Of the questionnaires sent, 573 (7.1%) were returned because of an incorrect or outdated address, and no updated address could be found. In total, 5,039 controls completed and returned the questionnaire, representing 62.1% of control subjects ascertained. Because there was no case with salivary gland cancer in Prince Edward Island and age range for cases with salivary gland cancer was from 26 to 76, the controls in Prince Edward Island and controls younger than 26 were excluded from this analysis. In each sex within each province, controls in some age groups (5-year age group) without corresponding cases in the same 5-year age group were also excluded from this analysis. Therefore, 3076 controls were included in the current study.

### 2.2. Data Collection

The registries used the same protocol to collect data from cases and control subjects, which was through the administration of a mailed questionnaire. For inadequately completed questionnaires, telephone follow-up was attempted for clarification and completeness, according to a structured protocol.

The mailed questionnaire collected information on demographic factors and other potential cancer risk factors, including education, family income, ethnicity, marital status, height, weight, alcohol consumption, recreational physical activity, diet, and vitamin and mineral supplement use two years before the interview. In addition, the questionnaire gathered information on smoking history, reproductive history, exposure to specific occupational chemicals, lifetime employment history, and Canadian residential history.

### 2.3. Assessment of Dietary Intake

The questionnaire used a 69-item food frequency instrument to collect information on diet two years before the interview date. The questionnaire also collected information regarding general changes in diet compared to 20 years previous, as well as vitamin or mineral supplement use during the previous 20 years. The design of the diet component of the questionnaire was based on two validated instruments: the National Cancer Institute's Block Questionnaire [11] and the instrument used in the Nurses' Health Study cohort [12], with minor modifications to account for the differences between the Canadian and American diet. These two instruments have been widely used in studies on diet and cancer.

A commonly used portion or serving size was specified for each food or beverage item. Respondents indicated the usual frequency of consumption of that portion size for each food item by selecting one of nine possible categories: 0 or <1 per month, 1 to 3 per month, 1 per week, 2 to 4 per week, 5 to 6 per week, 1 per day, 2 to 3 per day, 4 to 5 per day, and ≥6 per day. The weekly consumption of each food item was then calculated as the product of frequency and serving size. The nutrient content of foods was determined using food composition data from the Nutrient Value of Some Common Foods [13]. The total weekly intake level of each nutrient was determined by multiplying the weekly consumption quantity of each food item by its associated nutrient value and calculating the sum of the weekly nutrient intake levels for all 69 food items.

The questionnaire also collected information regarding vitamin or mineral supplement use (frequency and length) during the past 20 years. Respondents were asked how often (never, not regularly, and fairly regularly) and for how many years in total they had taken each of the 10 vitamins and mineral supplements (less than 1 year, 1-2 years, 3–5 years, 6–9 years, 10–19 years, and 20+ years). The ten vitamins and minerals are multiple vitamins, vitamin A, vitamin C, vitamin E, B-complex vitamins, beta carotene, calcium, iron, zinc, and selenium.

### 2.4. Assessment of Other Variables

The questionnaire gathered information on recreational physical activity 2 years before interview. Respondents were asked in which seasons, how often, and how long per session, on average, they participated in each of the 12 most common types of leisure-time physical activity in Canada. Individual activities included walking for exercise, bowling or curling, social dancing, gardening or yard work, golf, home exercise or exercise class, bicycling, jogging or running, racquet sports, swimming or water exercise, skiing or skating, and other strenuous exercises. Respondents indicated their usual frequency of participating in each of the above activities by choosing one of the following categories: never, less than once per month, 1–3 times per month, 1-2 times per week, 3–6 times per week, or every day.

As a measure of overweight and obesity, body mass index (BMI) was calculated as the reference weight in kilograms (2 years before interview) divided by height in meters squared. Subjects were classified as three categories: 18.5–<25.0, 25.0–<30.0, and ≥30.0.

Chemical exposure was defined as having ever worked at work or home with any of the following: asbestos, arsenic salts, chromium salts, cadmium salts, asphalt, mineral oils, benzidine, benzene, isopropyl oil, dyestuffs, vinyl chloride, pesticides, herbicides, mustard gas, welding, or wood dust.

### 2.5. Statistical Analysis

Analyses were conducted to evaluate the risks of salivary gland cancer associated with different potential risk factors. Odds ratios (ORs) and corresponding 95% confidence intervals (CIs) were computed using unconditional logistic regression modeling with the software package SAS (version 9.2). Dietary factors of interest were categorized by tertile or other appropriate cut-off points. The tertile cut-off points were based on the distribution in the control population.

Because controls were matched to the overall 19-cancer case group rather than the salivary gland cancer cases, all analyses were adjusted for age (years, continuous) and province of residence to account for the uneven distribution of these variables between the two groups. Macronutrients and food intakes were also adjusted for total energy intake. All trends tests were conducted by treating the different categories as a single ordinal variable.

## 3. Results

Of the 132 cases, 41 cases (31.1%) were adenoid cystic carcinoma, 26 cases (19.7%) were mucoepidermoid carcinoma, 17 (12.9%) were acinic cell carcinoma, 11 (8.3%) were squamous cell carcinoma, 10 (7.6%) were adenocarcinoma not otherwise specified, and the remaining 27 cases were various other histologic types.

Table 1 shows the distribution of some selected characteristics of salivary gland cancer cases and controls. Compared with controls, cases were younger and drank more alcohol. There were no significant differences in mean value for BMI, frequency of total recreational physical activity, total calorie intake, and smoking pack years between cases and controls.

Table 2 gives the ORs and 95% CIs for salivary gland cancer associated with family income, education, smoking, BMI, physical activity, calorie intake, alcohol consumption, coffee and tea consumption, occupational exposure to radiation, and chemical exposure. Compared with people with 0–8 years of education, those with a higher than high school education had an adjusted OR of 0.65 (95% CI: 0.40–1.06) for salivary gland cancer. A nonstatistically significantly increased OR for salivary gland cancer was found among obese (BMI ≥ 30) people compared with normal weight (BMI: 18.5–<25.0) people and among individuals with more than seven drinks per week of alcohol compared with those drinking no alcohol, among people with 40 or more pack years of smoking compared with those never smoked, and those people with occupational exposure to radiation compared with those never exposed. In addition, compared to people with less than 8 sessions/month of recreational physical activity, those with 8–≤30 and >30 sessions/month had ORs and 95% CIs of 0.85 (0.56–1.29) and 0.78 (0.50–1.23), respectively.

Table 3 shows the ORs and 95% CIs for salivary gland cancer associated with intakes of some food groups. Individuals with ≥4 servings/week intakes of processed meat were at an increased OR as compared with those with less than 1.4 servings/week intake (OR = 1.62, 95% CI: 1.02–2.58). Compared with people with less than 0.5 cup/week consumption of spinach/squash, those with more than 1.5 cups/week consumption had an adjusted OR of 0.62 (95% CI: 0.38–1.01). People with high intake of all vegetables and vegetable juices also had a nonsignificantly decreased OR (0.75, 95% CI: 0.47–1.20). However, we did not find any significant relationship of salivary gland cancer risk with consumption of dairy product, total vegetables, cruciferous vegetable, all fruits, all fruit and juice, all cereals, all breads, all rice/pasta, all whole grain produce, all grain produce, nut produce, fresh red meat, baked desserts, and margarine/butter/mayonnaise.

Table 4 demonstrates the ORs and 95% CIs associated with intakes of some macronutrients. Protein, carbohydrate, total fat, saturated fat, monounsaturated fat, polyunsaturated fat, trans fat, cholesterol, and total fiber were all showed to be not associated with the risk of salivary gland cancer.

Table 5 shows the ORs and 95% CIs for salivary gland cancer associated with intakes of vitamin and mineral supplements. The results indicated that years of taking multiple vitamins, vitamin A, vitamin C, vitamin E, B-complex vitamins, zinc, beta carotene, calcium, and iron were found to be not statistically significantly associated with the risk of salivary gland cancer.

## 4. Discussion

Although the statistical power of the current study is limited, this study suggested that high consumption of processed meat, obesity, more than 7 drinks/week of alcohol, and occupational exposure to radiation were associated with an increased risk of salivary gland cancer. Our study also suggested that high education level (>12 years) and high consumption of spinach/squash and vegetables/vegetable juices as a group were associated with a decreased risk of salivary gland cancer. However, these increases or decreases were not statistically significant except for processed meat.

Our finding of a significantly increased risk with processed meat consumption has yet not been reported by other studies, because none have specifically reported on it. Of the three dietary studies, one did not assess the association between meat consumption and salivary gland cancer [14], while the other two found no significant association with meat consumption but did not specifically examine processed meat consumption [15, 16]. The AICR/WCRF expert panel concluded in 2007 that the link between diets high in red and processed meat and colorectal cancer is convincing and every 50 gram serving of processed meat (roughly equivalent to 1 hot dog) eaten per day increases colorectal cancer risk by 21 percent [17]. The large European Prospective Investigation into Cancer and Nutrition observed a moderate positive association of processed meat consumption with all-cause mortality and mortalities of cardiovascular diseases, overall cancer [18], and colorectal cancer [19]. Processed meat contains nitrates and nitrites and is a major source of N-nitroso compounds (NOCs) and NOC precursors [20]. Nitrites or nitrates (e.g., sodium nitrite) added to meat for preservation can also form NOCs endogenously in animals and humans, and NOCs have been proved to be carcinogenic [21, 22]. Nitrites and nitrates from processed meat intake have been shown to increase the risk of colorectal adenoma, and higher level of intake of nitrite and nitrate was associated with an increased risk of colorectal adenoma compared with no or low level of intake [23]. Experiments have shown that NOCs induced parotid gland tumors in laboratory mice [24]. Higher incidence of parotid gland cancer has been found in workers of rubber industry in which nitroso compounds were used [25].

Our finding of an inverse association with consumption of vegetables, especially spinach and squash, corroborates the findings of two other studies [14, 15]. Vegetables are rich in a variety of nutrients, including vitamins, trace minerals, dietary fiber, and many other classes of biologically active compounds (e.g., phytochemicals) [26, 27]. These phytochemicals work in additive and synergistic way to exhibit antioxidant and anticancer activities [26–28]. Dietary spinach contains active compounds such as natural antioxidant mixture which has been shown to have tumor suppressive effects in carcinogenesis induced by a heterocyclic amine from cooked meat [27, 29].

Our study also observed a nonsignificant increased risk of salivary gland cancer associated with obesity. Only two studies have examined BMI and salivary gland cancer. A hospital-based case-control study of 128 cases and 114 controls reported a nonsignificantly decreased OR associated with low BMI compared with high BMI in men [30]. A study on 224 cases of salivary gland tumors (58 were malignant) and 214 nontumor controls (undergoing dental surgery) found that 45.5% cases were obese compared with 17.7% obese controls (*p* < 0.001) [31]. Although this relation needs to be confirmed by additional larger studies, our study provides further evidence that salivary gland cancer is associated with obesity.

In addition, we found that higher alcohol consumption was associated with a nonsignificant increase for salivary gland cancer risk. Most studies which have looked at this relationship have found no association [15, 30, 32–34], while a case-control study of 64 salivary gland carcinoma and 127 patient controls observed a significantly increased risk among women [35]. One case-control study on genetic variation in MDM2 (murine double minute-2) and* p*14^ARF^ and susceptibility to salivary gland carcinoma (SGC) found that the association of high-risk genotypes of MDM2 and* p*14^ARF^ with SGC risk was pronounced among ever-drinkers compared to that among nondrinkers, suggesting a greater susceptibility to SGC in ever-drinkers [36]. MDM2 and* p*14^ARF^ are the principal cellular regulators of p53 in response to stressors including radiation exposure and exposure to various chemical agents [37].

The increased risk of salivary gland cancer among those with a high number of pack years of smoking indicated in our study is consistent with some, but not all studies. Several studies have indicated a positive association between smoking and salivary gland cancer [33, 38–40]. The largest study until now (459 cases and 1265 population controls) found an increased risk of parotid gland tumors associated with cigarette smoking, and the trends of increasing risk were observed with increasing smoking intensity, pack years, and smoking duration [38]. This study also found a remarkably high risk for Warthin tumor with OR of 15.3 (95% CI: 6.1–38.5) for ever-cigarette smoker, an increased risk for malignant tumors (OR = 1.69, 95% CI: 0.81–3.51), and no association for pleomorphic adenoma (OR = 1.01, 95% CI: 0.75–1.37). However, other studies showed no association [30, 32, 34, 35, 41]. A strong association with smoking has been demonstrated consistently for benign Warthin tumors [38, 42–48] although most of these studies had a relative small sample size. The inconsistent results on the association between smoking and salivary gland cancer could be in part due to whether Warthin's tumor cases were included or the proportion of cases of Warthin's tumor in these studies. Our study and most other studies that showed no association included no cases of Warthin's tumor. However, a greater susceptibility to SGC in smokers was suggested in the study on genetic variation in MDM2 and* p*14^ARF^ and susceptibility to SGC [36]. Other studies also found that genetic polymorphisms of some other genes in combination with smoking conferred greater susceptibility to head and neck cancers [49–51].

This study has a number of limitations. This study is limited by the small number of cases. The response rate was low among cases, which may have resulted in selection bias. Among population controls, respondents may have had more favourable health-related characteristics compared to nonrespondents, such as higher levels of education or income. Exposure misclassification may have also occurred if subjects' responses were influenced by their beliefs about cancer and some lifestyle factors. This misclassification would likely be nondifferential because little is known about the etiology of salivary gland cancer. Exposure misclassification may have also occurred because the etiologically relevant time period for salivary gland cancer development is unknown. As a result, the data collected may not accurately reflect subjects' information during the etiologically relevant time period. The reference date used in this study was two years prior to the time of interview.

In conclusion, our population-based study suggested that high consumption of processed meat, obesity, heavy alcohol consumption, and occupational exposure to radiation were possible risk factors for salivary gland cancer. This study also suggested the possible role of high education level and consumption of vegetables, particularly spinach and squash, in reducing the risk of salivary gland cancer. However, the small number of cases and multiple comparisons preclude strong conclusions; therefore, further studies are warranted to increase the confidence in and to confirm our findings.

## Figures and Tables

**Table 1:** Characteristics of cases for salivary gland cancer and controls, Canada, 1994–1997.

Variable	Cases	Controls
Age (years) (mean ± SD)	56.6 ± 13.2	59.0 ± 12.6
26–29 (%)	2.3	1.5
30–34 (%)	5.3	3.5
35–39 (%)	9.1	4.2
40–44 (%)	5.3	6.1
45–49 (%)	4.6	8.7
50–54 (%)	11.4	6.0
55–59 (%)	14.4	10.4
60–64 (%)	12.9	16.8
65–69 (%)	15.9	19.9
70–76 (%)	18.9	22.9
BMI (kg/m^2^) (mean ± SD)	26.2 ± 4.5	25.7 ± 4.6
Alcohol consumption (drinks/week) (mean ± SD)	7.2 ± 10.6	5.4 ± 10.0
Smoking pack years (mean ± SD)	16.1 ± 20.8	16.1 ± 22.1
Total calorie intake (kcal/week) (mean ± SD)	13652 ± 5050	13284 ± 5870
Total recreational physical activity (sessions/month) (mean ± SD)	20.1 ± 17.9	21.4 ± 18.7
Province of residence (%)		
Newfoundland and Labrador	0.8	0.5
Nova Scotia	4.6	5.9
Ontario	68.9	61.6
Manitoba	3.8	2.4
Saskatchewan	3.0	2.2
Alberta	7.6	9.1
British Columbia	11.4	18.4
Marital status (%)		
Never married	9.9	5.6
Ever married (including common law)	90.1	94.4
Sex (%)		
Male	56.1	52.8
Female	43.9	47.2

**Table 2:** Odds ratios (OR) of salivary gland cancer associated with some demographic and lifestyle factors.

Variables	Cases	Controls	Adjusted^*∗*^		
			OR	95% CI	*p* trend
Family income adequacy					
Low income	14	454	Ref		0.29
Lower middle income	21	482	1.17	0.69–1.98	
Upper middle income	35	748	1.19	0.76–1.86	
High income	28	545	1.28	0.79–2.08	
Education (years)					
0–8	30	558	Ref		0.12
9–12	48	1249	0.67	0.41–1.07	
>12	52	1258	0.65	0.40–1.06	
Smoking status					
Never smoked	51	1164	Ref		0.87
Ex-smoker	51	1254	0.96	0.63–1.47	
Current smoker	30	658	0.97	0.60–1.54	
Smoking pack years					
0 (never smoked)	51	1164	Ref		0.54
1–<20	36	880	0.91	0.59–1.42	
20–<40	21	558	0.88	0.52–1.49	
≥40	23	443	1.35	0.78–2.33	
Body mass index (kg/m^2^)					
18.5–<25.0	58	1406	Ref		0.17
25.0–<30.0	47	1173	0.97	0.65–1.45	
≥30.0	26	409	1.52	0.94–2.45	
Moderate and strenuous recreational physical activity (sessions/month)					
<8	50	1015	Ref		0.29
8–30	46	1093	0.85	0.56–1.29	
>30	36	968	0.78	0.50–1.23	
Total energy intake (kcalories/week)					
<10747	36	1022	Ref		0.47
10747–<14429	52	1028	1.44	0.93–2.23	
≥14429	44	1022	1.19	0.76–1.88	
Alcohol consumption (drinks/week)					
0	43	1124	Ref		0.23
1–7	52	1287	0.99	0.65–1.50	
>7	37	663	1.37	0.85–2.20	
Coffee consumption (average cups/week)					
0–7	56	1270	Ref		0.93
>7–17.5	49	1143	0.98	0.66–1.45	
>17.5	27	600	0.98	0.61–1.58	
Tea consumption (average cups/week)					
0	31	729	Ref		0.30
1–7	56	1301	1.09	0.70–1.72	
>7	43	943	1.29	0.79–1.20	
Occupational exposure to radiation					
No	120	2913	Ref		
Yes	10	160	1.39	0.72–2.72	
Chemical exposure^*∗∗*^					
No	81	1934	Ref		
Yes	51	1142	1.01	0.68–1.48	

^*∗*^Adjusted for age, sex, and province of residence.

^*∗∗*^Worked at work or home with asbestos, arsenic salts, chromium salts, cadmium salts, asphalt, mineral oils, benzidine, benzene, isopropyl oil, dyestuffs, vinyl chloride, pesticides, herbicides, mustard gas, welding, and wood dust.

**Table 3:** Odds ratios of salivary gland cancer associated with some food groups.

Variable	Cases	Controls	Adjusted^*∗*^		
			OR	95% CI	*p* for trend
Dairy product (servings/week)					
<7	39	1018	Ref		0.27
7–<13.5	41	1015	1.04	0.66–1.63	
≥13.5	52	1041	1.28	0.82–2.03	
Tomatoes + carrots + broccoli (0.5 cups/week)					
<3.9	46	1048	Ref		0.49
3.9–7	51	1004	1.23	0.81–1.86	
>7	35	1022	0.83	0.52–1.33	
Spinach + squash (0.5 cups/week)					
<0.5	40	743	Ref		0.06
0.5–1.5	60	1333	0.85	0.56–1.29	
>1.5	32	998	0.62	0.38–1.01	
All yellow-green vegetables (0.5 cups/week)^#^					
<5	46	1016	Ref		0.61
5–<10	48	1023	1.11	0.73–1.70	
≥10	38	1035	0.88	0.56–1.39	
Cruciferous vegetable (0.5 cups/week)^*µ*^					
<1	41	938	Ref		0.50
1–<3.5	42	1072	0.93	0.60–1.45	
≥3.5	49	1064	1.16	0.75–1.79	
All vegetables (servings/week)					
<14.5	50	1010	Ref		0.54
14.5–22.5	40	1045	0.81	0.52–1.25	
>22.5	42	1019	0.87	0.55–1.38	
All vegetables and vegetable juices (servings/week)					
<15	51	1014	Ref		0.23
15–<24	43	1036	0.85	0.56–1.31	
≥24	38	1024	0.75	0.47–1.20	
All fruits (servings/week)					
<6.9	41	1007	Ref		0.40
6.9–<13.4	44	1039	1.11	0.71–1.72	
≥13.4	47	1028	1.21	0.78–1.90	
Apple, banana & other fruits (servings/week)					
<4.5	40	931	Ref		0.31
4.5–<9.5	40	1087	0.91	0.58–1.43	
≥9.5	52	1056	1.25	0.80–1.94	
All juices (4 oz/week)					
<3	42	984	Ref		0.70
3–<9	46	1063	0.98	0.64–1.51	
≥9	44	1027	0.92	0.59–1.43	
All fruit & juice (servings/week)					
<12.4	44	1018	Ref		0.65
12.4–<22.5	39	1022	0.93	0.60–1.45	
≥22.5	49	1034	1.11	0.71–1.73	
All cereals (cups/week)					
≤1	46	1040	Ref		0.43
>1–<6.5	50	1007	1.14	0.76–1.73	
≥6.5	36	1027	0.81	0.51–1.30	
All breads (slices/week)					
≤7	51	1267	Ref		0.26
>7–≤17.5	38	978	1.00	0.65–1.55	
>17.5	48	829	1.31	0.84–2.05	
All rice and pasta (cups/week)					
≤1.5	41	1213	Ref		0.17
>1.5–4	56	1141	1.41	0.93–2.15	
>4	35	720	1.38	0.85–2.24	
All whole grain product (servings/week)					
≤3.5	43	1025	Ref		0.40
>3.5–<11.5	42	1013	1.05	0.68–1.63	
≥11.5	47	1036	1.21	0.78–1.90	
All grain product (servings/week)^e^					
≤15	39	1011	Ref		0.24
>15–<25.5	42	1019	1.11	0.70–1.74	
≥25.5	51	1045	1.33	0.82–2.15	
Tofu & lentil (servings/week)					
0	47	1250	Ref		0.37
<2	74	1531	1.35	0.93–1.97	
≥2	11	293	1.04	0.53–2.05	
Nut (servings/week)					
<0.5	51	1302	Ref		0.67
0.5–≤2	47	810	1.43	0.95–2.15	
>2	34	962	0.86	0.55–1.35	
Fresh red meat (servings/week)					
<2.5	40	922	Ref		0.46
2.5–<4.5	43	955	0.98	0.63–1.53	
≥4.5	49	1197	0.85	0.54–1.33	
Processed meat (servings/week)					
<1.4	33	989	Ref		0.03
1.4–<4	37	1037	1.02	0.63–1.66	
≥4	62	1048	1.62	1.02–2.58	
All meat (servings/week)					
<4.9	35	1020	Ref		0.36
4.9–<9.3	47	1030	1.26	0.60–1.98	
≥9.3	50	1024	1.26	0.78–2.05	
Baked desserts (servings/week)					
<1.9	45	1076	Ref		0.57
1.9–6.5	48	982	1.15	0.76–1.74	
>6.5	39	1016	0.86	0.54–1.37	
Margarine, butter & mayonnaise (servings/week)					
<6	42	944	Ref		0.37
6–<16.5	38	1099	0.79	0.50–1.24	
≥16.5	52	1031	1.22	0.78–1.89	

^*∗*^Adjusted for age, sex, province of residence, and total energy intake.

#: tomatoes + carrots + broccoli + spinach + squash + sweet potato.

*µ*: broccoli + cabbage.

e: all cereals + all breads + all rice and pasta.

**Table 4:** Odds ratios of salivary gland cancer associated with some macronutrients.

Variable	Cases	Controls	Adjusted^*∗*^		
			OR	95% CI	*p* trend
Protein (g/week)					
1st tertile	41	1023	Ref		0.59
2nd tertile	42	1027	1.01	0.64–1.61	
3rd tertile	49	1022	1.67	0.67–2.02	
Carbohydrate (g/week)					
1st tertile	36	1023	Ref		0.54
2nd tertile	52	1027	1.45	0.92–2.29	
3rd tertile	44	1022	1.18	0.66–2.11	
Total fiber (g/week)					
1st tertile	46	1022	Ref		0.97
2nd tertile	42	1028	0.94	0.60–1.46	
3rd tertile	44	1022	0.99	0.60–1.65	
Total fat (g/week)					
1st tertile	42	1022	Ref		0.56
2nd tertile	49	1028	1.10	0.71–1.70	
3rd tertile	41	1022	0.84	0.49–1.44	
Saturated fat (g/week)					
1st tertile	41	1023	Ref		0.52
2nd tertile	41	1026	0.99	0.62–1.56	
3rd tertile	50	1023	1.20	0.70–2.04	
Monounsaturated fat (g/week)					
1st tertile	41	1022	Ref		0.80
2nd tertile	49	1028	1.16	0.75–1.80	
3rd tertile	42	1022	0.92	0.53–1.58	
Polyunsaturated fat (g/week)					
1st tertile	42	1023	Ref		0.74
2nd tertile	50	1026	1.16	0.75–1.80	
3rd tertile	40	1023	0.89	0.52–1.54	
Trans fat (g/week)					
1st tertile	43	1023	Ref		1.00
2nd tertile	47	1026	1.08	0.70–1.66	
3rd tertile	42	1022	1.00	0.61–1.62	
Cholesterol (g/week)					
1st tertile	41	1023	Ref		0.55
2nd tertile	42	1027	1.02	0.65–1.58	
3rd tertile	49	1022	1.16	0.71–1.91	

^*∗*^Adjusted for age, sex, province of residence, and total energy intake.

**Table 5:** Odds ratios of salivary gland cancer associated with some vitamin/mineral supplements.

Variable	Case	Control	Adjusted^*∗*^		
			OR	95% CI	*p* for trend
Multiple vitamin (years taken)					
Never	55	1504	Ref		0.16
<4 years	33	649	1.34	0.85–2.12	
≥4 years	44	923	1.35	0.88–2.06	
Vitamin A (years taken)					
Never	106	2570	Ref		0.23
<4 years	12	247	1.20	0.65–2.21	
≥4 years	14	259	1.38	0.78–2.46	
Vitamin C (years taken)					
Never	58	1491	Ref		0.70
<4 years	38	665	1.46	0.95–2.24	
≥4 years	36	920	1.05	0.68–1.61	
Vitamin E (years taken)					
Never	78	1904	Ref		0.41
<4 years	26	530	1.23	0.78–1.94	
≥4 years	28	642	1.16	0.74–1.82	
Beta carotene (years taken)					
Never	112	2653	Ref		1.00
<4 years	14	227	1.53	0.86–2.73	
≥4 years	6	196	0.74	0.32–1.70	
B-complex vitamins (years taken)					
Never	94	2309	Ref		0.20
<4 years	17	372	1.14	0.66–1.95	
≥4 years	21	395	1.38	0.84–2.27	
Calcium (years taken)					
Never	89	2066	Ref		0.54
<4 years	20	466	1.09	0.65–1.82	
4 years	23	542	1.16	0.71–1.90	
Iron (years taken)					
Never	101	2356	Ref		0.81
<4 years	19	400	1.10	0.65–1.88	
≥4 years	12	320	0.87	0.46–1.63	
Zinc (years taken)					
Never	113	2690	Ref		0.93
<4 years	14	195	1.72	0.96–3.07	
≥4 years	5	191	0.64	0.26–1.58	

^*∗*^Adjusted for age, sex, and province of residence.
